# Complete Genome Sequence of *Corynebacterium pseudotuberculosis* Strain E19, Isolated from a Horse in Chile

**DOI:** 10.1128/genomeA.01385-15

**Published:** 2015-11-25

**Authors:** Ana Lídia Q. Cavalcante, Larissa M. Dias, Jorianne T. C. Alves, Adonney A. O. Veras, Luis C. Guimarães, Flávia S. Rocha, Alfonso Gala-García, Patricio Retamal, Rommel T. J. Ramos, Vasco Azevedo, Artur Silva, Adriana R. Carneiro

**Affiliations:** a Federal University of Pará, Center of Genomic and Systems Biology, Laboratory of Genomics and Bioinformatics, Belém, Pará, Brazil; b Institute of Biological Sciences, Federal University of Minas Gerais, Belo Horizonte, Minas Gerais, Brazil; c Department of Animal Preventive Medicine, Faculty of Veterinary and Animal Sciences, University of Chile, Santiago, Chile

## Abstract

*Corynebacterium pseudotuberculosis* is related to several diseases infecting horses and small ruminants, causing economic losses to agribusiness. Here, we present the genome sequence of *C. pseudotuberculosis* strain E19. The genome includes one circular chromosome 2,367,956 bp (52.1% G+C content), with 2,112 genes predicted, 12 rRNAs, and 48 tRNAs.

## GENOME ANNOUNCEMENT

*Corynebacterium pseudotuberculosis* is a pathogenic Gram-positive bacterium that belongs to a suprageneric group of actinomycetes (1), which also includes the genera *Corynebacterium*, *Mycobacterium*, *Nocardia*, and *Rhodococcus*, termed the CMNR group. This group has particular characteristics, such as a cell wall organization composed mainly of peptidoglycan, arabinogalactan, and mycolic acids (2, 3). The meso-diaminopimelic acid (meso-DAP) present in peptidoglycan is related to the wall’s resistance against most peptidases contributing to bacteria dissemination (2).

The biochemical property reduction of nitrate to nitrite is used to distinguish *C. pseudotuberculosis* biovars: *equi* (nitrate positive) and *ovis* (nitrate negative) (2, 3). This species has a wide host range with different degrees of severity (4).

*C. pseudotuberculosis* biovar *equi* is the etiological agent of ulcerative lymphangitis (UL), commonly called “pigeon fever” (5). UL has several symptoms, however, the most common clinical manifestation of this disease is characterized by external pectoral or ventral abscesses (6).

*C. pseudotuberculosis* strain E19 (biovar *equi*) was isolated from the cervical abscess of a horse in Patagonia, Magallanes Region, Chile. Genome sequencing was performed using the Ion Torrent PGM sequencer (Life Technologies) with a fragments library.

The semiconductor sequencing revealed 557,925,027 pb with a coverage of 243×. The quality of the raw data was analyzed using the web tool FastQC (http://www.bioinformatics.babraham.ac.uk/projects/fastqc/). Reads with Phred 20 were assembled using a *de novo* strategy with SPAdes 3.1.0 software (7). The assembly produced a total of 40 contigs with *N*_50_ of 259,252. Additionally, a scaffold was generated using Mauve software (8), using *C. pseudotuberculosis* strain 258 (accession no. CP003540). The gap closure was performed using CLC Genomics Workbench 8 (http://www.clcbio.com/) and Artemis (9), generating a contig of 2,367,956 pb size.

The genome was automatically annotated using Rapid Annotations using Subsystem Techonology (RAST) (10). The manual curation of the annotation was performed using Artemis software (9) and the nonredundant (nr) protein database of the National Center for Biotechnology Information (NCBI) and UniProt (http://www.uniprot.org). CLC Genomics Workbench 8 software was used to correct indel errors in homopolymer regions. rRNAs and tRNAs were predicted using RNAmmer (11) and tRNAScan-SE (12) software, respectively.

The genome includes one circular chromosome of 2,367,956 bp (52.1% G+C content), and 2,112 genes were predicted, of which 565 (26.7%) were classified as hypothetical proteins, with 12 rRNAs and 48 tRNAs; 10 pseudogenes were also identified.

### Nucleotide sequence accession number.

The genome project has been deposited in GenBank under the accession number CP012136.
